# Effects of Vitamin C and/or E Supplementation on Glycemic Control and Cardiovascular Risk Factors in Type 2 Diabetes: A Systematic Review and Subgroup Meta-analysis

**DOI:** 10.1093/nutrit/nuaf133

**Published:** 2025-08-05

**Authors:** Jerónimo Aragón-Vela, Jesús R Huertas, Rafael A Casuso

**Affiliations:** Department of Health Sciences, Area of Physiology, University of Jaén, 23071 Jaén, Spain; Institute of Nutrition and Food Technology, Department of Physiology, University of Granada, 18071 Granada, Spain; Department of Health Sciences, Universidad Loyola Andalucía, 14004 Córdoba, Spain

**Keywords:** HDL, systolic blood pressure, ascorbic acid, alpha-tocopherol

## Abstract

**Context:**

Type 2 diabetes (T2D) is one of the fastest-growing global health emergencies of the 21st century. However, high antioxidant capacity of supplementation of vitamin C and/or E was inversely associated with insulin resistance. However, each antioxidant possesses a distinct biological function that may be influenced by both dosage and duration of supplementation, potentially resulting in significantly different effects.

**Objective:**

This meta-analysis aimed to evaluate whether vitamin C, vitamin E, or their combination is more effective in improving glycemic control, blood lipids, and blood pressure in individuals with T2D.

**Data Sources and Data Extraction:**

A systematic search was conducted in PubMed, Scopus, and Web of Science databases for randomized clinical trials, identifying 52 studies (n = 1425 participants).

**Data Analysis:**

Random-effects models were used to assess the effects of vitamin C and/or E supplementation on glycemic control, blood lipid levels, and blood pressure. These findings indicate that supplementation with vitamin C, vitamin E, or their combination has a comparable effect on glycemic index values, systolic blood pressure, and blood lipid profiles. However, a significant reduction in systolic blood pressure was observed only with vitamin C and combined vitamin C + E supplementation. Additionally, a significant increase in high-density-lipoprotein (HDL) levels was noted exclusively with the combined vitamin C + E supplementation.

**Conclusion:**

Consequently, supplementation with vitamin C, vitamin E, and their combination (C + E) exhibited differing effects on HDL levels and systolic blood pressure. However, their effects on glycemic control, diastolic blood pressure, and blood lipids other than HDL were comparable.

**Systematic Review Registration:**

PROSPERO registration no. CRD42023399366

## INTRODUCTION

The findings of the 10th edition of the *International Diabetes Federation Diabetes Atlas* confirmed that diabetes mellitus (DM) is one of the fastest-growing global health emergencies of the 21st century.^1^ It is estimated that, by 2045, DM will affect 783 million people.^1^ Diabetes mellitus is characterized by chronic hyperglycemia resulting from insulin resistance and defects in insulin secretion and/or action caused by islet β-cell failure.^2^ Additionally, several comorbidities are associated with insulin resistance, including obesity, hypertension, cardiovascular disorders, and dyslipidemia.^3^ Beyond these conditions, numerous studies have highlighted oxidative stress alterations as central to the development of type 2 diabetes (T2D).^4^^,^^5^ Specifically, oxidative stress increases insulin resistance through mechanisms such as inhibition of insulin signaling and the promotion of inflammatory processes.^4^ Consistent with these findings, a high antioxidant capacity from supplementation has been inversely associated with insulin resistance.^6^ However, each antioxidant exhibits distinct biological functions that may vary depending on the dosage and duration of supplementation, potentially resulting in significantly different effects.

Vitamin C (ascorbic acid), a water-soluble antioxidant, has been therapeutically investigated in patients with T2D.^7^ The meta-analysis conducted by Mason et al^6^ suggested that supplementation with vitamin C may be effective in improving glycemic control and blood pressure in this population. Furthermore, vitamin C plays a role in regenerating vitamin E.^2^

Vitamin E, a lipophilic antioxidant, comprises a group of compounds including α-, β-, γ-, and δ-tocopherol, with α-tocopherol being the most abundant and biologically active.^2^ Several studies have demonstrated that vitamin E supplementation provides benefits in T2D by inhibiting the formation of advanced glycosylation end-products^8^^,^^9^ and mitigating the long-term effects of lipid peroxidation on pancreatic β-cell dysfunction.^10^ Both vitamins have been extensively tested in diabetic patients. Nonetheless, there is a paucity of in-depth comparative studies assessing which of these 2 vitamins offers greater benefits to this population.

Therefore, both vitamin C and vitamin E contain essential micronutrients, primarily obtained through the diet but also widely available as over-the-counter supplements, offering a common and accessible adjunctive treatment for concomitant insulin-resistant oxidative stress in T2D.

Accordingly, the main objective of this study was to conduct a systematic review and meta-analysis of randomized controlled trials (RCTs) comparing the effectiveness of vitamins C and/or E in improving glycemic control, blood lipids, and blood pressure in people with T2D.

## METHODS

### Eligibility and Search Strategy

The study methodology followed the Preferred Reporting Items for Systematic Reviews and Meta-Analyses (PRISMA) 2020 statement.^11^ The protocol was registered in the International Prospective Register of Systematic Reviews (PROSPERO) database (CRD42023399366). The Scopus, PubMed, and Web of Science databases were systematically searched for eligible articles published up to May 31, 2024. The following search strategy was used: (“vitamin*” or “vitamin C” or “ascorbic acid” or “vitamin E” or “tocopherol”) and (“HbA1c” or “glycosylated haemoglobin” or “glucose” or “cholesterol” or “LDL” or “HDL” or “Triacylglycerol” or “triglycerides” or “blood lipids” or “blood pressure” or “hypertension” or “systolic blood pressure” or “diastolic blood pressure” or “mean blood pressure”) and (“type 2 diabetes” or “obesity” or “insulin resistance” or “metabolic syndrome”). Studies were included if they contained the key words (as mentioned above) and met the PICOS (Participants, Intervention, Comparator, Outcomes, Study design) criteria (**Table 1**). To identify missing studies, each selected study was individually scrutinized by clicking on the “cited” and “similar” tabs of the databases.

**Table 1.** PICOS Criteria for Inclusion and Exclusion of Studies

Parameter	Inclusion criteria	Exclusion criteria
Participants	Adults (≥18 y) with T2D	Animal and in vitro studies
Intervention	RCTs of at least 2 wk of vitamin C and/or vitamin E supplementation	Co-supplementation with other substances
Comparators	Placebo vs intervention (baseline and endpoint)	Intermediate time points
Outcomes	Concentrations of LDL, HDL, TC, HbA1c, SBP, DBP, FBS, and insulin	Studies not reporting concentrations of LDL, HDL, TC, HbA1c, SBP, DBP, FBS, and insulin
Study design	Randomized clinical trials, crossover studies	Reviews, systematic reviews, letters, case reports or case series, abstracts, publications without full text

Abbreviations: DBP, diastolic blood pressure; FBS, fasting blood sugar; HbA1c, glycated hemoglobin; HDL, high-density lipoprotein; LDL, low-density lipoprotein; RCT, randomized controlled trial; SBP, systolic blood pressure; T2D, type 2 diabetes; TC, total cholesterol; TG, total triglycerides.

### Study Selection

**Figure 1.**
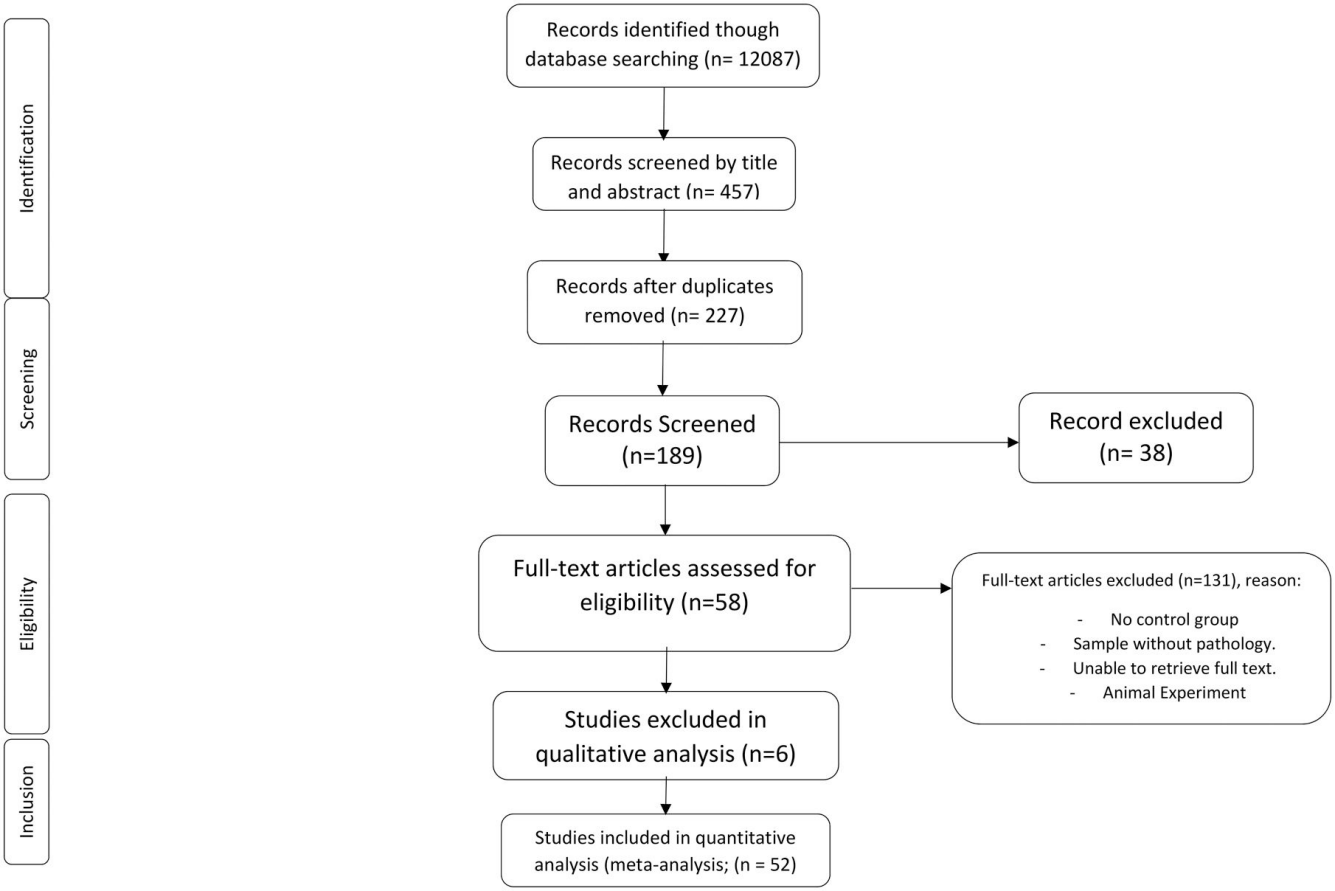
Flow Diagram of the Study Selection Procedure Showing Eligible Randomized Controlled Trials for Meta-analysis

The selection of studies was independently performed by 2 reviewers (R.A.C. and J.A.-V.). Studies were first screened based on their titles and abstracts. Discrepancies during study selection were resolved by consensus and/or by the opinion of a third author (J.R.H.). The original search yielded 12 087 studies (Scopus = 9584, Web of Science = 1312, PubMed = 1191), 84 of which were independently read and reviewed after deduplication and screening. A total of 52 studies were included in the qualitative and quantitative analyses (**Figure 1**). Studies involving animal subjects were excluded, as were those incorporating other forms of supplementation or additional physical activity along with vitamin C and/or E supplementation. In addition, only studies published in English or Spanish were eligible for analysis. In terms of the inclusion criteria, eligibility was limited to studies that included a control or placebo group. Furthermore, only studies focusing on participants diagnosed with DM were considered, and 3 studies were included in the analysis.

The following data were independently extracted by 2 researchers (R.A.C. and J.A.-V.): number of participants, age, body mass index (BMI), duration of diabetes, type of vitamins, dosage, duration of supplementation, glycated hemoglobin (HbA1c), fasting blood glucose (FBG), insulin, homeostatic model assessment of insulin resistance (HOMA-IR), diastolic blood pressure (DBP), systolic blood pressure (SBP), high-density lipoprotein (HDL), low-density lipoprotein (LDL), triglycerides (TG), and total cholesterol (TC) (**Table 1**). Where data were not presented in the text or tables and the authors could not be reached, data were extracted using WebPlot Digitizer (version 4.6, Ankit Rohatgi, Pacifica, CA, USA).^12^ Any discrepancies between reviewers were resolved by consensus.

Four articles including patients with type 1 diabetes were identified. Fuller et al^13^ had an experimental group consisting of 9 patients with insulin-dependent diabetes (IDD) and 6 with non-IDD (NIDD) and a control group consisting of 4 patients with IDD and 9 with NIDD. In the study by Khatami et al,^14^ both the control and experimental groups had 10% of participants with IDD (3 individuals), while the remaining 90% had NIDD (27 individuals). Likewise, Aghadavod et al^15^ included 11% of participants with IDD in both control and experimental groups. Economides et al^16^ analyzed the effects of vitamin E supplementation in patients with IDD and those with NIDD; however, we only recorded data from patients with NIDD. Therefore, the overall analysis included data from 20 patients with IDD.

Studies conducted by Ellulu et al^17^ and Bishop et al,^18^ despite vitamin C supplementation, were excluded from our meta-analysis because they included hypertensive participants and/or diabetic obese adults without further clarification. In addition, studies carried out by Nayaka^19^ and Mohammadi et al^20^ were excluded because the authors presented their data without SD values.

### Data Synthesis and Analysis

All analyses were conducted using the metafor package in R software (R Foundation for Statistical Computing, Vienna, Austria).^21^ Meta-analyses were performed using random-effects models with DerSimonian–Laird methods to compare the effects of vitamin C and/or E supplementation on glycemic index, blood lipids, and blood pressure. Mean and SD variations were recorded separately for each study, intervention, and measured outcome. If a change in SD was not provided, we followed a previously used methodology.^6^^,^^22^^,^^23^ In this case, we calculated the SD change assuming a correlation coefficient of 0.7 as follows:


$$\Delta \mathrm{SD}=\sqrt{({\mathrm{SD}}_{\mathrm{pre}}^{2}+{\mathrm{SD}}_{\mathrm{post}}^{2}-2\times \mathrm{corr}\times {\mathrm{SD}}_{\mathrm{pre}}\times {\mathrm{SD}}_{\mathrm{post}}).}$$

Effect sizes are presented as the mean difference (MD) and 95% CIs or standardized mean difference (SMD) and 95% CIs when the outcome had noncomparable scales. A subgroup analysis was performed to directly compare the effects of studies using different vitamin supplements.

### Heterogeneity and Risk of Bias

Heterogeneity between studies was assessed using Cochran’s *Q* and *I^2^* statistics, and the prediction interval derived from tau. Publication bias was assessed by visual inspection of funnel plots for asymmetries, and Egger’s test was used to quantify publication bias by analyzing funnel plot asymmetries.

The evaluation of bias in the studies (R.A.C. and J.A.-V.) was conducted using the Critical Appraisal Checklist for Case-Control Studies or RCT, developed by the Faculty of Health and Medical Sciences at the University of Adelaide, South Australia.^24^ This checklist includes 8–13 criteria covering various sections of research articles, such as the title, abstract, introduction, methods, results, and discussion. However, because not all criteria were relevant to every study, quality scores were determined both as a total point sum and as a percentage of the applicable items.

## RESULTS

### Study Characteristics

The details of the participants and outcomes are detailed in **Table S1**. The original search yielded 1046 studies; after duplication and screening, 189 studies were independently read and reviewed. A total of 52 studies were included in this meta-analysis. Participant details and outcomes are presented in **Table S1**. A total of 25 studies (*n* = 679 participants) evaluated vitamin C supplementation.^25–49^ We also identified 25 studies (680 participants) that evaluated vitamin E supplementation.^13–16^^,^^36^^,^^49–68^ Finally, 5 studies (*n* = 66 participants) evaluated vitamin E and C supplementation.^8^^,^^9^^,^^49^^,^^69^^,^^70^ The supplementation protocols across all included studies lasted from 2 to 48 weeks. A risk-of-bias analysis indicated that all studies were of medium to high quality (**Table S2**).

#### Effects of Vitamin E and/or Vitamin C on Glycemic Control

**Figure 2.**
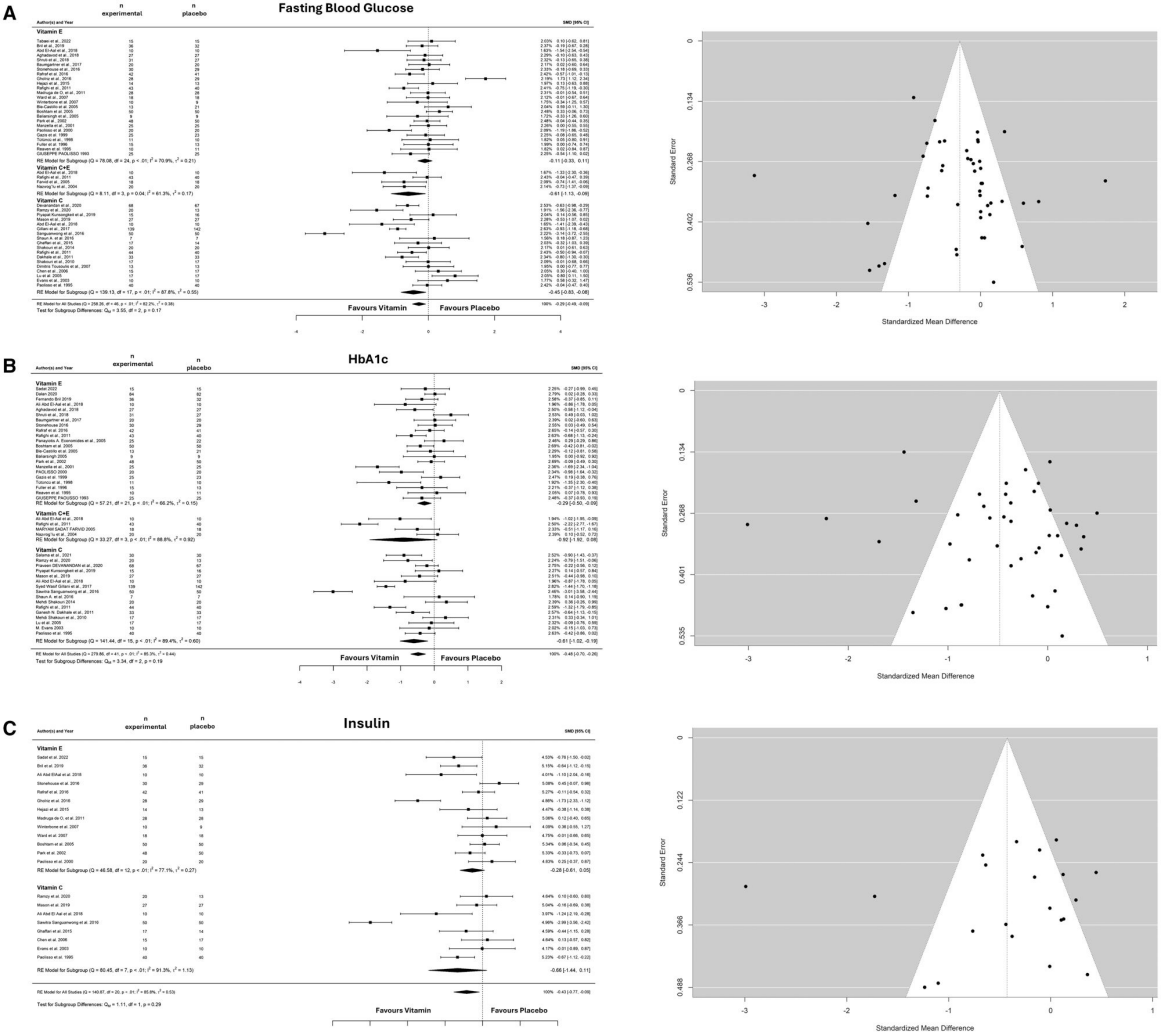
Forest and Funnel Plot of the General Effects and the Subgroup Analysis (Vitamins C, E, and C + E) on Fasting Blood Glucose (A) HbA1c (B), and (C) Insulin. Abbreviations: HbA1c, glycated hemoglobin; RE, random effects; SMD, standardized mean difference

The overall effect of the supplementation (vitamin C, vitamin E, and vitamins C + E) was to reduce FBG levels (SMD: −0.287; 95% CI: −0.487 to −0.088; *I^2^* = 82%, *P* = .004; *n* = 47 studies) (**Figure 2A**). No funnel plot asymmetries were revealed after applying the Egger’s test for FBG levels (*P* = .262) (**Figure 2A**). Subgroup analysis revealed no differences between supplementation strategies (*P* = .17). However, it is important to note that the isolated analysis revealed that vitamin E did not reduce FBG levels (*P* = .345) (**Figure 2A**).

For HbA1c, the model showed an overall reduction following supplementation (SMD: −0.482; 95% CI: −0.705 to −0.259; *I^2^* = 85%, *P* < .001; *n* = 42 studies) (**Figure 2B**). Egger’s test revealed no funnel plot asymmetry (*P* = .488) (**Figure 2B**). Subgroup analysis revealed no differences between the supplementation groups (*P* = .188). However, while the isolated effect of supplementation with vitamin C or E was to reduce HbA1c levels, the combination of vitamin C + E did not reduce it (*P* = .073).

Insulin levels were assessed in only 21 studies (*n* = 8 vitamin C and *n* = 13 vitamin E) (**Figure 2C**). The overall effect was a significant reduction in insulin levels (SMD: −0.427; 95% CI: −0.769 to −0.085; *I^2^* = 85%, *P* = .014; *n* = 20 studies) (**Figure 2C**). No funnel plot asymmetries were observed after applying the Egger’s test for insulin (*P* =0.60) (**Figure 2C**). Subgroup analysis showed a similar effect between supplementations (*P* = .0292). When analyzed in isolation, neither vitamin C (*P* = .094) nor vitamin E (*P* = .101) reduced the insulin levels.

**Figure 3.**
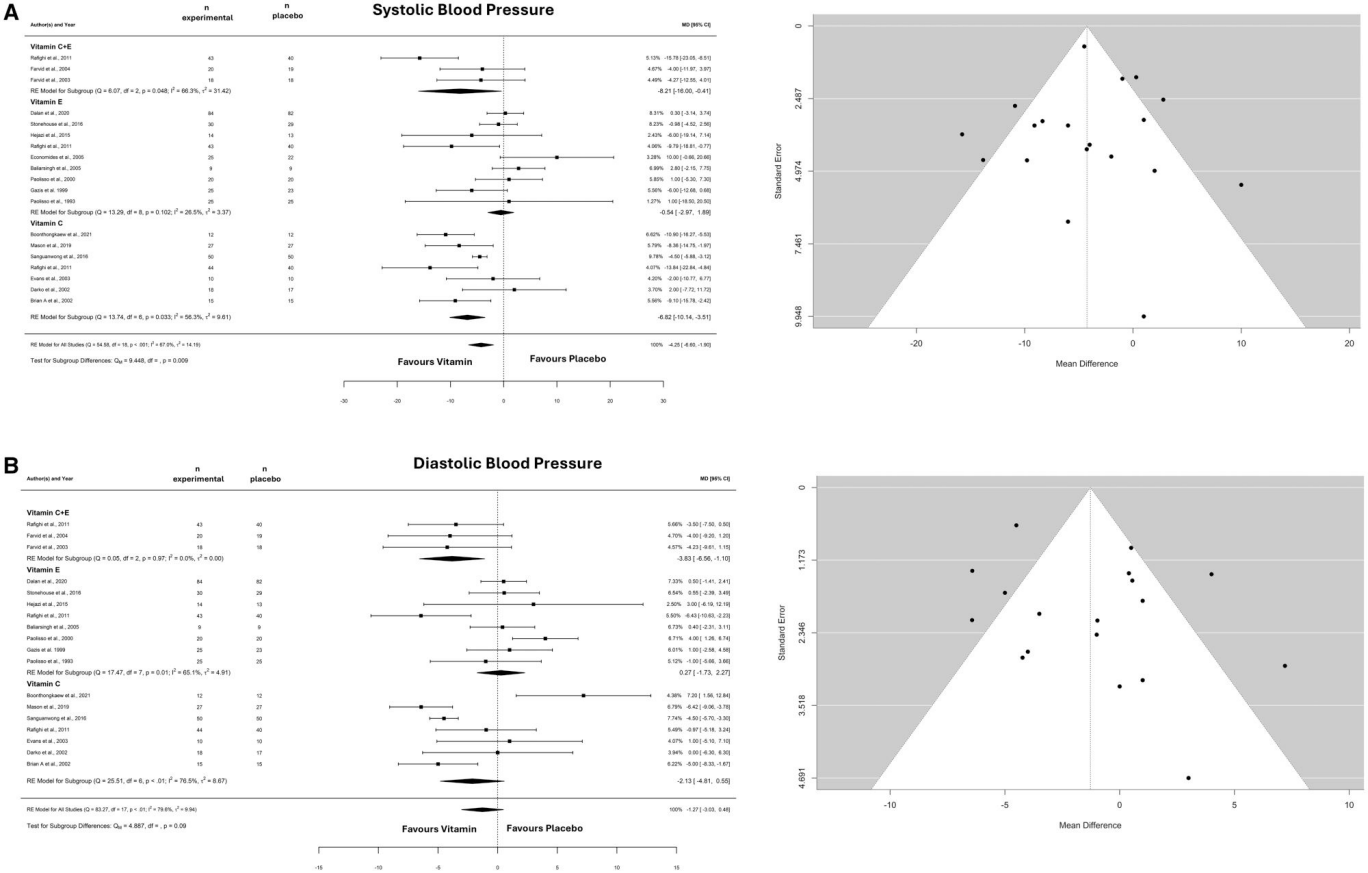
Forest and Funnel Plot of the General Effects and the Subgroup Analysis (Vitamins C, E, and C + E) on Systolic (A) and Diastolic (B) Blood Pressure. Abbreviation: MD, mean difference

With regard to the effects of vitamin E and/or C on blood pressure the model showed an overall significant reduction in SBP (mmHg: −4.252; 95% CI: −6.600 to −1.903; *I^2^* = 67%, *P* = .0004; *n* = 19 studies) (**Figure 3A**). Egger’s test revealed no funnel plot asymmetry (*P* = .887) (**Figure 3A**). The subgroup analysis revealed a significant difference (*P* = .010) between the supplements. Indeed, only vitamin C (*P* < .0001) and vitamins C + E (*P* = .039) reduced SBP.

The overall effect of DBP was not significant (mmHg: −1.274; 95% CI: −3.025 to 0.477; *I^2^* = 80%, *P* = 0.154) (**Figure 3B**). Egger’s test revealed no funnel plot asymmetry (*P* = .173) (**Figure 3B**). Subgroup analysis was not significant (*P* = .161). However, the only intervention able to reduce DBP was a combination of vitamins C and E (*P* = .006).

#### Effects of Vitamin E and/or Vitamin C on Blood Lipids

**Figure 4.**
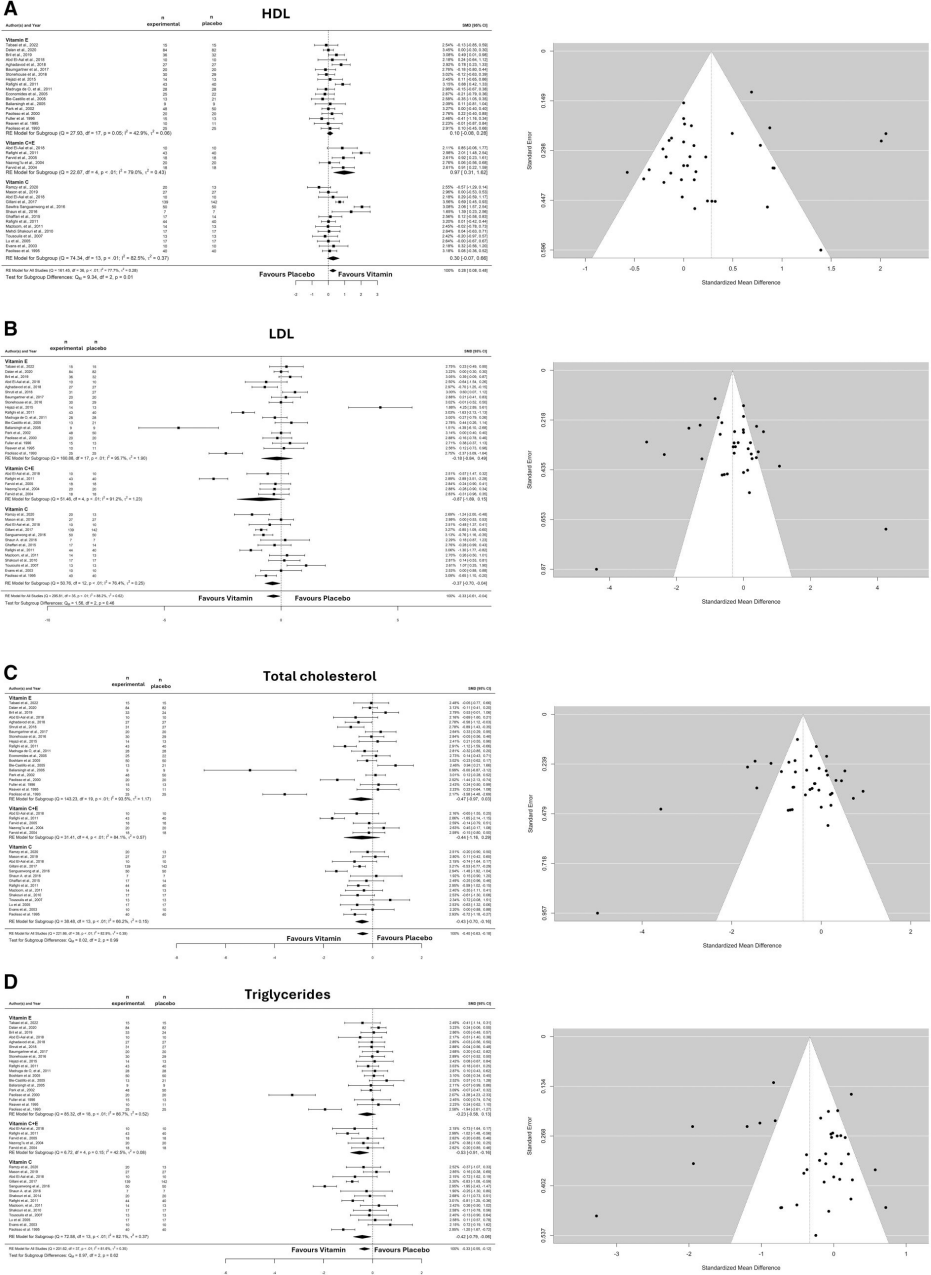
Forest and Funnel Plot of the General Effects and the Subgroup Analysis (Vitamin C, E, and C + E) on HDL (A), LDL (B), Total Cholesterol (C), and Triglycerides (D). Abbreviations: HDL, high-density lipoprotein; LDL, low-density lipoprotein; SMD, standardized mean difference

The analysis revealed an overall HDL increase following supplementation (SMD: 0.256; 95% CI: 0.064–0.453; *I^2^* = 77%, *P* = .009; *n* = 39 studies) (**Figure 4A**). Egger’s test showed no significant funnel plot asymmetry (*P* = .301) (**Figure 4A**). The subgroup analysis was significant for different supplements (*P* = .01). The only intervention able to increase HDL levels was vitamin C + E supplementation (*P* = .003).

LDL levels were reduced overall (SMD: −0.325; 95% CI: −0.61 0.043; *I^2^* = 88%, *P* = .024; *n* = 36 studies) (**Figure 4B**). Egger’s test showed no funnel plot asymmetry (*P* = .383) (**Figure 4B**). Subgroup analysis showed no differences between the supplementation groups (*P* = .459). However, the isolated analysis revealed that vitamin C was the only supplement able to reduce LDL levels (*P* = .028).

The overall analysis showed a reduction in TC levels (SMD: −0.404; 95% CI: −0.62, −0.181; *I^2^* = 83%, *P* = .0004; *n* = 39 studies) (**Figure 4C**). Egger’s test showed no funnel plot asymmetry (*P* = .82) (**Figure 4C**). The subgroup analysis revealed no differences between the supplements (*P* = .990). The isolated analysis showed that vitamin C reduced TC (*P* = .002) and vitamin C + E levels (*P* = .096).

The overall model showed that TG levels were reduced following supplementation (SMD: −0.331; 95% CI: −0.54 to −0.11; *I^2^* = 82%, *P* = .0027; *n* = 38 studies) (**Figure 4D**). Egger’s test revealed no funnel plot asymmetry (*P* = .636) (**Figure 4D**). Subgroup analysis showed no significant differences between the interventions (*P* = .616). Isolated analysis showed that vitamin C (*P* = .023) and vitamins C + E (*P* = .006) were the only interventions able to reduce TG levels.

## DISCUSSION

The primary aim of this study was to conduct a subgroup meta-analysis to compare the efficacy of vitamin C, vitamin E, or their combination in managing cardiometabolic health in participants with T2D. We found a synergistic effect of combining vitamins C and E, with increments in HDL levels. However, when analyzing blood pressure, we found a similar efficacy between consuming vitamin C or a combination of vitamins C and E. For the remaining cardiometabolic outcomes, no clear differences were observed between supplementation strategies.

A previous meta-analysis^71^ reported that, in patients with dyslipidemia, vitamin C supplementation can decrease LDL and TG levels without affecting HDL levels. Another meta-analysis showed that, in a broader sample (including both healthy and diseased populations), vitamin E can increase HDL without affecting LDL or TG levels.^72^ Therefore, depending on the pathophysiological state, different interventions may have a greater impact, which is the key to precision medicine. Our data showed that vitamin C may have a greater impact on lowering blood lipids in diabetic individuals, whereas vitamin E showed no evidence of a meaningful effect. Contradictory findings have been reported in several studies, where vitamin C supplementation showed no significant benefits in lipid-related variables such as TC, TG, LDL, and HDL.^29^^,^^35^^,^^41^^,^^43^ These results may be attributed to the relatively short duration of the supplementation protocols. Indeed, these studies typically implemented interventions lasting between 2 and 6 weeks, despite using relatively high doses ranging from 1 to 2 g per day. However, Shakouri Mahmoudabadi et al^45^ demonstrated that a lower dose of 200 mg per day administered over a longer period of 8 weeks resulted in reductions in TC, LDL, and TG levels, along with an increase in HDL levels. In addition, Mohammad et al^73^ and our findings emphasize the ineffectiveness of vitamin E in enhancing lipid profiles. Nevertheless, in line with our results, although only in the male population, Salonen et al^74^ showed that vitamin C tends to raise HDL, whereas vitamin E does not. However, a clear advantage for the increase in HDL levels was observed when consuming a combination of vitamin C and E supplementation. In clinical practice, vitamin C supplementation alone may be recommended for diabetic patients with normal or mildly altered lipid profiles to support the maintenance of LDL, TG, and TC within physiological levels. However, if the therapeutic goal is to increase HDL levels, combining vitamins C and E appears to be the more effective strategy.

Our findings also demonstrate that supplementation with vitamin C and the combination of vitamins C + E leads to a significant reduction in SBP, whereas supplementation with vitamin E alone does not induce any measurable changes. However, various lines of evidence suggest that vitamin E may play a critical role in the prevention of cardiovascular diseases, particularly coronary heart disease and atherosclerosis.^75^ Vitamin E exists in several homologues—α-, β-, γ-, and δ-tocopherols—with α-tocopherol showing the highest antioxidant capacity and, consequently, receiving the most scientific attention.^46^ Nonetheless, γ-tocopherol possesses distinct biological functions, notably its unique ability to neutralize reactive nitrogen species, whose accumulation contributes to cellular damage and death.^76^ Despite its lower bioavailability and bioactivity compared with α-tocopherol, γ-tocopherol's specific chemical reactivity, metabolism, and additional properties—such as anti-inflammatory and natriuretic effects—underscore its potential complementary role in cardiovascular protection.^46^

However, with regard to the effects of vitamin C on vascular function, recent mechanistic studies have provided supporting evidence for the biological plausibility of its role in reducing SBP.^6^^,^^77^ Potential mechanisms through which vitamin C may exert hypotensive effects involve its ability to enhance the synthesis and bioavailability of nitric oxide (NO) through antioxidant actions.^78^ It has been hypothesized that vitamin C may neutralize superoxide, thereby reducing NO's reactivity of NO with superoxide and limiting the formation of peroxynitrite, a reactive species that can damage the vasculature.^79^ Additionally, vitamin C helps preserve tetrahydrobiopterin levels, a cofactor required for endothelial NO synthase, thus supporting NO production via this enzyme.^80^^,^^81^ The lack of an effect observed with vitamin E has not been elucidated at the cellular level. It has been suggested that high circulating glucose levels can increase cyclooxygenase-2 in endothelial cells.^82^ In vitro studies have shown that vitamin E is not an efficient treatment to decrease cyclooxygenase-2 levels.^83^ Nevertheless, future studies are needed to understand the different roles of vitamins C and E in regulating endothelial physiology in patients with diabetes. Notably, we also found that the combination of vitamins C + E also decreased SBP; however, this should be interpreted with caution, as this observation was derived from only 3 studies. Therefore, vitamin C alone should be considered the preferred supplementation strategy to reduce blood pressure in diabetic patients.

This study has several strengths and limitations. The major limitation of the present meta-analysis is the significant heterogeneity observed among the included studies, including differences in supplement dosage, sample characteristics, timing of outcome measurements, and length of follow-up. Heterogeneity refers to the variability in effect estimates across studies due to differences in populations, interventions, or methodologies. Additionally, variables such as age, dose, and duration of supplementation were not examined through meta-regression. An additional limitation is the lack of information on plasma vitamin C concentrations at baseline and after supplementation in the majority of studies. Considering that vitamin C deficiency is frequently observed in individuals with poorly controlled diabetes, primarily due to enhanced urinary excretion, this omission may partly explain interindividual differences in response and contribute to outcome variability. Additionally, we recommend that future authors investigating the effects of α-tocopherol clearly specify whether the α-tocopherol used is synthetic or natural. This will allow for a more accurate comparison of dosages between trials.

However, the most important strength of this study is that the vast majority of the included trials were RCTs, allowing for more reliable inferences about causality. Furthermore, to the best of our knowledge, this is the first meta-analysis to directly compare the efficacy of both vitamins, including their combination.

## CONCLUSION

We observed a synergistic effect when vitamins C and E were combined, leading to increased HDL levels. However, with regard to blood pressure, the effectiveness of vitamin C alone was comparable to that of the combined supplementation of vitamins C and E. For the remaining cardiometabolic outcomes, no significant differences were detected between the different supplementation strategies.

## Supplementary Material

nuaf133_Supplementary_Data

## Data Availability

All the data are available in the manuscript or supplementary files.
