# Supplementary material for: Effects of Vitamin C and/or E Supplementation on Glycemic Control and Cardiovascular Risk Factors in Type 2 Diabetes: A Systematic Review and Subgroup Meta-analysis
Source: Nutr Rev. 2025 Aug 5;84(2):235–45. doi: 10.1093/nutrit/nuaf133 (PMC12793614; doi:10.1093/nutrit/nuaf133)
Supplement: nuaf133_Supplementary_Data [file nuaf133_supplementary_data.docx]

**Supplementary Material**

**Effects of Vitamin C and/or E Supplementation on Glycaemic Control and Cardiovascular Risk Factors in Type 2 Diabetes: A systematic review and subgroup meta-analysis.**

Jerónimo Aragón-Vela PhD ^1^; Jesús R. Huertas PhD ^3^; Rafael A. Casuso PhD^3,4^

Supplementary Table 1. Characteristics of the eligibles studies. Values are expressed as mean (SD).

| Author, Year | Country | Protocol | Studied Arms | Vitamin E used | Participants Age (y) | Sample Size: Vitamin/Placebo | Vitamin intervention Duration (Weeks) | Baseline BMI (kg/m2) | Outcome | Vitamin Dose |
| --- | --- | --- | --- | --- | --- | --- | --- | --- | --- | --- |
| Aghadavod et al., 2018 | Iran | RCT | Vit E /Placebo | Not specified | 63,3 (9,5) | 27/27 | 12 | 31 (5,4) | TC↓, LDL↓, HDL↑, TG→, FBS→ | Vit E - 800 IU/d |
| Ali Abd El-Aal et al., 2015 | Palestine | RCT | Placebo/ Vit C/vitamin E/ vitamin C + E | Not specified | 40-60 | 10/10/10/10 | 12 | 30,9 (1,21) | FBS↓, HbA1↓, insulin↓, HOMA-IR↓, TG↓, TC↓, LDL↓, HDL↓, BMI→, SBP→, DBP→ | Vit E - 400mg/vit C-500 mg |
| Baliarsingh et al., 2005 | India | RCT | Vit E /Placebo | α-tocopherol, α-tocotrienol, β-tocotrienol, γ-tocotrienol, δ-tocopherol and δ-tocotrienol | 50,5 (6,2) | (9/9 | 8 | 23,2 (2,3) | LDL↓, TC↓, SBP→, DBP→, FBS→, HbA1→HDL→, TG→ | Vit E - 190 mg/d |
| Bascil et al., 1998 | Turkey | RCT | Vit E /Placebo | Not specified | 58,2 (11,4) | 11-oct | 24 | 27,4 (5,6) | FBS→, HbA1→ | Vit E - 900 mg/d |
| Baumgartner et al., 2017 | Netherlands | RCT | Vit E /Placebo | α-tocopherol | 57 | 20/20 | 4 | 29,5 | FBS→, HbA1→, HDL→, LDL→, TC→, TG→ | Vit E - 804 mg/d |
| Ble-Castillo et al., 2005 | Mexico | RCT | Vit E /Placebo | α-tocopherol | 53,3 (12,8) | 13/21 | 6 | 27,5 (4,4) | SBP→, DBP→, BMI→, TG→, FBS→, HDL→, LDL→ | Vit E - 800 IU/d |
| Boonthongkaew et al., 2021 | Thailand | RCT | Vit C /Placebo | N/A | 53 (7) | (12/12 | 6 |  | FBS→, HbA1c→, SBP↓, DBP↓ | Vit C - 1 g/d |
| Boshtam et al., 2005 | Iran | RCT | Vit E /Placebo | Not specified | 53,6 (8,05) | 50/50 | 27 | 24,6 (3,6) | FBS→, Insulin→, HbA1→, TG→, TC→ | Vit E - 200 IU/d |
| Bril et al., 2019 | U.S. | RCT | Vit E /Placebo | Not specified | 58,5 (10) | 36/32 | 18 | 33,7 (4,3) | BMI→, FBS→, HbA1c→, TC↓, HDL↓, LDL→, TG→, Insulin↓ | Vit E - 400 IU/d |
| Chen et al., 2006 | U.S. | RCT | Vit C /Placebo | N/A | 50 (1) | 15/17 | 4 | 35 (1) | BMI→, TC→, TG→, FBS→, HbA1c→, Insulin→, HOMA-IR→ | Vit C - 800 mg/d |
| Dakhale et al., 2011 | India | RCT | Vit C /Placebo | N/A | 47,05 (1,4) | 33/33 | 12 |  | FBS↓, HbA1c↓ | Vit C - 1 g/d |
| Dalan et al., 2020 | Singapore | RCT | Vit E /Placebo | α-tocopherol | 56 (10) | 84/82 | 24 | 27,3 (4,94) | HbA1c→, HDL→, LDL→, TC→, BMI→, SBP→, DBP→ | Vit E- 268 mg/d* |
| Darko et al., 2002 | U.K. | RCT | Vit C /Placebo | N/A | 56,2 (1,6) | 18/17 | 3 | 29,1 (0,9) | HDL→, LDL→, TC→, BMI→, SBP→, DBP→, FBS→, TG→ | Vit C - 1,5 g/d |
| Devanandan et al., 2020 | India | RCT | Vit C /Placebo | N/A | 44,9 (7,7) | 68/67 | 36 | 23,7 (0,7) | FBS↓, HbA1c↓ | Vit C - 1 g/d |
| Economides et al., 2005 | U.S. | RCT | Vit E /Placebo | N/A | 59,9 (9) | 25/20 | 12 | 31,4 (6,7) | HbA1c→, HDL→, TC→ | Vit E - 1800 IU/d |
| Evans et al., 2003 | U.K. | CS | Vit C /Placebo | N/A | 52,5 (7,4) | 10-oct | 6 | 28,9 (5,1) | BMI→, TC→, LDL→, HDL↓, TG↓, SBP→, DBP→, insulin→, FBS→, HbA1c→ | Vit C - 1 g/d |
| Fuller et al.,1996 | U.S. | CS | Vit E /Placebo | α-tocopherol | 47 (13) | 15/15 | 8 | 27,05 (4,5) | TC→, TG→, LDL→, HDL→ | Vit E - 804 mg/d* |
| Gazis et al.,1999 | U.K. | CS | Vit E /Placebo | α-tocopherol | 56,1 (10,4) | 23/25 | 8 | 27,7 (3,9) | SBP↓, DBP→, BMI→, HbA1c↓, FBS↓ | Vit E - 1600 IU/d |
| Ghaffari et al., 2015 | Iran | CS | Vit C /Placebo | N/A | 50,9 (4,9) | 17/17 | 8 |  | FBS↓, insulin→, TC↓, HDL↓, LDL↓ | Vit C - 800 mg/d |
| Gholriz et al., 2016 | Iran | RCT | Vit E /Placebo | Not specified | 61,8 (11,5) | 30/30 | 12 | 30,5 (5,3) | insulin↓, HOMA-IR→, FBS→ | Vit E - 1200 IU/d |
| Hejazi et al., 2015 | Iran | RCT | Vit E /Placebo | Not specified | 47,3 (6,9) | 14/13 | 6 | 29,05 (5,51) | TG→, HDL→, LDL→, TC→, FBS→, insulin→, HOMA-IR→ | Vit E - 400 IU/d |
| Kunsongkeit et al., 2019 | Thailand | RCT | Vit C /Placebo | N/A | 58,8 (12,6) | 15/16 | 8 |  | FBS→, HbA1c→ | Vit C - 500 mg/d |
| Lu et al.,2005 | Sweden | RCT | Vit C /Placebo | N/A | 55 | 17/17 | 2 |  | FBS→, HbA1c→, TG→, TC→, HDL→ | Vit C - 1 g/d |
| Madruga et al., 2011 | Brazil | RCT | Vit E /Placebo | α-tocopherol | >60 | 25/26 | 4 | >27 | TC→, HDL→, LDL→, TG→, FBS→, insulin→, HOMA-IR→ | Vit E - 800 mg/d |
| Manzella et al., 2001 | Italy | RCT | Vit E /Placebo | α-tocopherol | 64,7 (4,3) | 25/25 | 16 | 26,3 (4,1) | FBS→, insulin↓, HOMA-IR↓, HbA1c↓ | Vit E - 600 mg/d |
| Mason et al., 2016 | Australia | RCT | Vit C /Placebo | N/A | 59,4 (3,5) | 07-jul | 16 | 30,7 (1,1) | Insulin↓, BMI→, FBS→, HbA1c→, TC→, LDL→, HDL→, TG→ | Vit C - 1 g/d |
| Mason et al., 2019 | Australia | RCT | Vit C /Placebo | N/A | 61,8 (6,8) | 27/27 | 16 | 29,1 (3,1) | BMI→, FBS↓, HbA1c→, insulin→, TC→, LDL→, HDL→, TG→, SBP↓, DBP↓, | Vit C - 1 g/d |
| Mazloom et al., 2011 | Iran | RCT | Vit C /Placebo | N/A | 46,8 (8,4) | 15/15 | 6 | 27,9 (4,16) | BMI→, FBS→, TG→, TC→, LDL→, HDL→ | Vit C- 1 g/d |
| Mullan et al., 2002 | Ireland | RCT | Vit C /Placebo | N/A | 59,2 (6,6) | 15/15 | 4 | 28,6 (4,3) | SBP↓, DBP↓ | Vit C- 500 mg/d |
| Nazıroglu et al., 2004 | Germany | CS | Vit E+C /Placebo | α-tocopherol | 51 | 20/20 | 6 |  | FBS↓, TC↓, HDL→, LDL↓, TG↓, HbA1c↓ | Vit E - 600mg/vit C-1 g/d |
| Paolisso et al., 1993 | Italy | RCT | Vit E /Placebo | α-tocopherol | 71,3 (0,8) | 25/25 | 12 | 27,3 | FBS↓, TC↓, LDL↓, HDL→, SBP→, DBP→, HbA1c↓, TG↓, insulin → | Vit E - 900 mg/d |
| Paolisso et al., 1995 | Italy | RCT | Vit C /Placebo | N/A | 72 (0,5) | 40/40 | 16 | 27,7 (0,4) | FBS↓, TC↓, LDL↓, HDL→, HbA1c↓, TG↓, insulin ↓ | Vit C - 1 g/d |
| Paolisso et al., 2000 | Italy | RCT | Vit E /Placebo | Not specified | 57,4 (5,9) | 20/20 | 8 | 27,3 (2,4) | FBS→, TC→, LDL→, HDL→, HbA1c→, TG→, insulin→, SBP→, DBP→, BMI→, | Vit E - 600 mg/d |
| Park et al., 2002 | Korea | RCT | Vit E /Placebo | α-tocopherol | 49,4 (9,8) | 48/50 | 8 | 23,2 (3,2) | BMI↑, FBS↓, HbAc1↓, insulin↓, TC↓, HDL→, LDL→, TG→ | Vit E - 200 mg/d |
| Rafighi et al., 2011 | Iran | CS | Vit C /Placebo | N/A | 52 | 44/40 | 12 | 29,7 | BMI→, FBS↓, HbA1c↓, TC↑, LDL↑, HDL↓, TG↑, SBP↓, DBP↓, | Vit C - 266,7 mg/d |
|  |  |  | Vit E+C/Vit C/Placebo | Not specified | 51 | 43/40 | 12 | 30,2 | BMI→, FBS↓, HbA1c↓, TC↑, LDL↑, HDL↓, TG↑, SBP↓, DBP↓, | Vit E - 300mg/d |
| Rafraf et al., 2016 | Iran | RCT | Vit E /Placebo | α-tocopherol | 53,75 (7,54) | 42/41 | 8 | 29,1 (3,60) | FBS↓, Hba1c↓, insulin↓, HOMA-IR↓ | Vit E - 400 IU/d |
| Ramzy et al., 2020 | Egypt | RCT | Vit C /Placebo | N/A | 56,4 (8,01) | 20/13 | 8 | 33,2 (5,8) | FBS→, HbA1c→, TC↓, TG→, HDL→, LDL↓, insulin→, HOMA-IR→ | Vit C - 500 mg/d |
| Reaven et al., 1995 | U.S. | CS | Vit E /Placebo | α-tocopherol | 61,3 (7,6) | (10/11 | 10 | 29,6 (4,2) | FBS→, insulin→, TC→, LDL→, TG→, HDL→ | Vit E - 1600 IU/d |
| Sadat et al., 2004 A | Iran | RCT | Vit E+C /group | Not specified | 49,7 (9,2) | 18/18 | 12 | 27,5 (4,1) | BMI→, SBP→, DBP→, | Vit E- 150 mg/Vit C- 200 mg/d |
| Sadat et al., 2004 B | Iran | RCT | Vit E+C /group | Not specified | 49,7 (9,2) | 18/18 | 12 | 27,5 (4,1) | TC→, HDL→, LDL→, TG→ | Vit E- 150 mg/Vit C- 200 mg/d |
| Sadat et al., 2005 | Iran | RCT | Vit E+C /group | Not specified | 50 (9) | 18/18 | 12 | 27,4 (4,1) | TC→, TG→, HDL→, LDL→, SBP→, DBP→, HbA1c→ | Vit E- 100 IU/Vit C- 200 mg/d |
| Sadat et al., 2022 | Iran | RCT | Vit E /Placebo | α-tocopherol | 50,5 (1,2) | 15/15 | 12 | 26,9 (0,7) | BMI→, FBS→, HbA1c↓, HDL→, LDL↓, TC↓, TG↓, insulin↓, HOMA-IR↓ | Vit E- 400 IU/d |
| Salama et al., 2021 | Egypt | RCT | Vit C /Placebo | N/A | 48,2 (7,2) | 30/30 | 12 | 31,9 (3,09) | BMI→, HbAc1↓ | Vit C - 1 g/d |
| Sanguanwong et al., 2016 | Thailand | RCT | Vit C /Placebo | N/A | 57,7 | 50/50 | 8 | 25,5 | BMI→, SBP→, DBP→, FBS↓, TC→, TG↓, HDL↑, LDL→, HbA1c↓, Insulin↓, HOMA-IR↓ | Vit C - 1 g/d |
| Shakouri et al., 2011 | Iran | RCT | Vit C /Placebo | N/A | 51,2 (6,8) | 17/17 | 8 | 29,1 (4,6) | FBS↓, HbA1c↓, TC↓, LDL↓, HDL↑, TG↓ | Vit C 200 mg/d |
| Shakouri et al., 2014 | Iran | CS | Vit C /Placebo | N/A | 51,5 (6) | 20/20 | 8 | 29 (4,5) | BMI→, FBS↓, HbA1c↓, TG↓ | Vit C 200 mg/d |
| Shruti et al., 2018 | India | RCT | Vit E /Placebo | Not specified | 51,5 (9,2) | 31/27 | 12 | 25,5 | BMI↑, FBS↓, HbA1c↓, TC→, TG→, LDL↓ | Vit E 400 mg/d |
| Stonehouse et al., 2016 | Australia | RCT | Vit E /Placebo | palm-tocotrienols | 60,5 | 28/29 | 8 | 33,8 | TC→, HDL→, LDL→, TG→, SBP→, DBP→, HbA1c→, insulin→, HOMA-IR→ | Vit E 552 mg/d |
| Tousoulis et al., 2007 | Greece | RCT | Vit C /Placebo | N/A | 60,1 (2,8) | 13/13 | 4 | 28,6 (0,8) | TC→, TG→, LDH→, HDL→, FBS→ | Vit C 2 g/d |
| Ward et al., 2007 | Australia | RCT | Vit E /Placebo | α-tocopherol, β-tocotrienol and δ-tocopherol | 63 (7) | 18/18 | 6 | 28,4 (3,1) | SBP↑, DBP→, FBS→, insulin→ | Vit E 500 mg/d |
| Wasif et al., 2017 | Saudi Arabia | RCT | Vit C /Placebo | N/A | 38 (8,02) | 139/142 | 48 | 23,9 (3,7) | BMI→, FBS↓, HbA1↓, LDL↓, HDL↓, TG↓, TC↓ | Vit C 500 mg/d |
| Winterbone et al.,2007 | U.K. | RCT | Vit E /Placebo | α-tocopherol | 62,3 (1,8) | (10/9 | 4 | 29,7 | FBS→, insulin→ | Vit E 1200 IU/d |

↑, increase; ↓ decrease; → no change; RDC, randomized double-blinded placebo controlled clinical trial; CS, control studies; TC, total cholesterol; LDL, low-density lipoprotein; HDL, high-density lipoprotein; TG, total triglycerides; FBS, Fasting blood sugar; SBP; Systolic blood pressure, DBP; diastolic blood pressure; HbA1c, glycated haemoglobin, BMI; body mass index. N/A = not applicable. * It has been converted from IU to mg as follows: 1 IU of the natural form is equivalent to 0.67 mg of alpha-tocopherol.

Supplementary Table 2. Checklist from Joanna Briggs Institute's criterium according to kind of study, percentage of criterium reached and quality level of evidence.

| Criteriums according to kind of study | | | | | | | | | | | | | | | |
| --- | --- | --- | --- | --- | --- | --- | --- | --- | --- | --- | --- | --- | --- | --- | --- |
| **Authors** | **1** | **2** | **3** | **4** | **5** | **6** | **7** | **8** | **9** | **10** | **11** | **12** | **13** | **Percentage reached** | **Quality level** |
| Aghadavod et al., 2018 | 1 | 1 | 1 | 1 | 1 | 1 | 1 | 1 | 1 | 1 | 1 | 1 | 1 | 100% | HQ |
| Ali Abd El-Aal et al., 2015 | 1 | 1 | 1 | 1 | 1 | 1 | 1 | 1 | 1 | 1 | 1 | 1 | 1 | 100% | HQ |
| Baliarsingh et al., 2005 | 1 | 1 | 1 | 1 | 1 | 1 | 1 | 1 | 1 | 1 | 1 | 1 | 1 | 100% | HQ |
| Bascil et al., 1998 | 1 | 1 | 1 | 1 | 1 | 1 | 1 | 1 | 1 | 1 | 1 | 1 | 1 | 100% | HQ |
| Baumgartner et al., 2017 | 1 | 1 | 1 | 1 | 1 | 1 | 1 | 1 | 1 | 1 | 1 | 1 | 1 | 100% | HQ |
| Ble-Castillo et al., 2005 | 1 | 0 | 1 | 0 | 0 | 0 | 1 | 1 | 1 | 1 | 1 | 1 | 1 | 69% | MQ |
| Boonthongkaew et al., 2021 | 1 | 1 | 1 | 1 | 1 | 1 | 1 | 1 | 1 | 1 | 1 | 1 | 1 | 100% | HQ |
| Boshtam et al., 2005 | 1 | 1 | 1 | 1 | 1 | 1 | 1 | 1 | 1 | 1 | 1 | 0 | 1 | 92% | HQ |
| Bril et al., 2019 | 1 | 1 | 1 | 1 | 1 | 1 | 1 | 1 | 1 | 1 | 1 | 1 | 1 | 100% | HQ |
| Chen et al., 2006 | 1 | 1 | 1 | 1 | 1 | 1 | 1 | 1 | 1 | 1 | 1 | 1 | 1 | 100% | HQ |
| Dakhale et al., 2011 | 1 | 1 | 1 | 1 | 1 | 1 | 1 | 1 | 1 | 1 | 1 | 1 | 1 | 100% | HQ |
| Dalan et al., 2020 | 1 | 1 | 1 | 1 | 1 | 1 | 1 | 1 | 1 | 1 | 1 | 1 | 1 | 100% | HQ |
| Darko et al., 2002 | 1 | 1 | 1 | 1 | 1 | 1 | 1 | 1 | 1 | 1 | 1 | 1 | 1 | 100% | HQ |
| Devanandan et al., 2020 | 1 | 1 | 1 | 0 | 0 | 0 | 1 | 1 | 1 | 1 | 1 | 1 | 1 | 77% | HQ |
| Economides et al., 2005 | 1 | 1 | 1 | 1 | 1 | 1 | 1 | 1 | 1 | 1 | 1 | 0 | 1 | 92% | HQ |
| Evans et al., 2003 | 1 | 0 | 1 | 1 | 1 | 0 | 0 | 1 | 1 | 1 |  |  |  | 70% | MQ |
| Fuller et al.,1996 | 1 | 1 | 1 | 1 | 1 | 0 | 1 | 1 | 1 | 1 |  |  |  | 90% | HQ |
| Gazis et al.,1999 | 1 | 1 | 1 | 1 | 1 | 0 | 1 | 1 | 1 | 1 |  |  |  | 90% | HQ |
| Ghaffari et al., 2015 | 1 | 1 | 1 | 1 | 1 | 0 | 1 | 1 | 1 | 1 |  |  |  | 90% | HQ |
| Gholriz et al., 2016 | 1 | 1 | 1 | 1 | 1 | 1 | 1 | 1 | 1 | 1 | 1 | 1 | 1 | 100% | HQ |
| Hejazi et al., 2015 | 1 | 1 | 1 | 1 | 1 | 1 | 1 | 1 | 1 | 1 | 1 | 0 | 1 | 92% | HQ |
| Kunsongkeit et al., 2019 | 1 | 1 | 1 | 1 | 1 | 1 | 1 | 1 | 1 | 1 | 1 | 0 | 1 | 92% | HQ |
| Lu et al.,2005 | 0 | 1 | 1 | 1 | 1 | 1 | 1 | 1 | 0 | 1 | 1 | 1 | 1 | 84% | HQ |
| Madruga et al., 2011 | 1 | 1 | 1 | 1 | 1 | 1 | 1 | 1 | 1 | 1 | 1 | 1 | 1 | 100% | HQ |
| Manzella et al., 2001 | 0 | 1 | 1 | 1 | 1 | 1 | 1 | 1 | 1 | 1 | 1 | 1 | 1 | 92% | HQ |
| Mason et al., 2016 | 1 | 1 | 1 | 1 | 1 | 1 | 1 | 1 | 1 | 1 | 1 | 1 | 1 | 100% | HQ |
| Mason et al., 2019 | 1 | 1 | 1 | 1 | 1 | 1 | 1 | 1 | 1 | 1 | 1 | 1 | 1 | 100% | HQ |
| Mazloom et al., 2011 | 0 | 0 | 1 | 1 | 1 | 1 | 1 | 1 | 1 | 1 | 1 | 0 | 1 | 77% | HQ |
| Mullan et al., 2002 | 1 | 1 | 1 | 1 | 1 | 1 | 1 | 1 | 1 | 1 | 1 | 1 | 1 | 100% | HQ |
| Nazıroglu et al., 2004 | 1 | 1 | 1 | 1 | 1 | 1 | 0 | 1 | 1 | 1 |  |  |  | 90% | HQ |
| Paolisso et al., 1993 | 0 | 1 | 1 | 1 | 1 | 1 | 1 | 1 | 1 | 1 | 1 | 1 | 1 | 92% | HQ |
| Paolisso et al., 1995 | 0 | 1 | 1 | 1 | 1 | 1 | 1 | 1 | 1 | 1 | 1 | 1 | 1 | 92% | HQ |
| Paolisso et al., 2000 | 0 | 1 | 1 | 1 | 1 | 1 | 1 | 1 | 1 | 1 | 1 | 1 | 1 | 92% | HQ |
| Park et al., 2002 | 0 | 1 | 1 | 0 | 0 | 0 | 1 | 1 | 1 | 1 | 1 | 0 | 1 | 61% | MQ |
| Rafighi et al., 2011 | 1 | 1 | 1 | 1 | 1 | 0 | 0 | 1 | 1 | 1 |  |  |  | 80% | HQ |
| Rafraf et al., 2016 | 1 | 1 | 1 | 1 | 1 | 1 | 1 | 1 | 1 | 1 | 1 | 1 | 1 | 100% | HQ |
| Ramzy et al., 2020 | 1 | 1 | 1 | 0 | 0 | 0 | 1 | 1 | 1 | 1 | 1 | 1 | 1 | 77% | HQ |
| Reaven et al., 1995 | 1 | 1 | 1 | 1 | 1 | 1 | 0 | 1 | 1 | 1 |  |  |  | 90% | HQ |
| Sadat et al., 2004 A | 0 | 1 | 1 | 1 | 1 | 1 | 1 | 1 | 1 | 1 | 1 | 1 | 1 | 92% | HQ |
| Sadat et al., 2004 B | 0 | 1 | 1 | 1 | 1 | 1 | 1 | 1 | 1 | 1 | 1 | 1 | 1 | 92% | HQ |
| Sadat et al., 2005 | 0 | 1 | 1 | 1 | 1 | 1 | 1 | 1 | 1 | 1 | 1 | 1 | 1 | 92% | HQ |
| Sadat et al., 2022 | 1 | 1 | 1 | 1 | 1 | 1 | 1 | 1 | 1 | 1 | 1 | 1 | 1 | 100% | HQ |
| Salama et al., 2021 | 1 | 1 | 1 | 1 | 1 | 1 | 1 | 1 | 1 | 1 | 1 | 1 | 1 | 100% | HQ |
| Sanguanwong et al., 2016 | 1 | 1 | 1 | 1 | 1 | 1 | 1 | 1 | 1 | 1 | 1 | 0 | 1 | 92% | HQ |
| Shakouri et al., 2011 | 0 | 1 | 1 | 0 | 0 | 1 | 1 | 1 | 1 | 1 | 1 | 0 | 1 | 70% | MQ |
| Shakouri et al., 2014 | 1 | 1 | 1 | 1 | 1 | 0 | 1 | 1 | 1 | 1 |  |  |  | 90% | HQ |
| Shruti et al., 2018 | 1 | 1 | 1 | 0 | 0 | 0 | 1 | 1 | 1 | 1 | 1 | 1 | 1 | 77% | HQ |
| Stonehouse et al., 2016 | 1 | 1 | 1 | 1 | 1 | 1 | 1 | 1 | 1 | 1 | 1 | 1 | 1 | 100% | HQ |
| Tousoulis et al., 2007 | 0 | 1 | 1 | 1 | 1 | 1 | 1 | 1 | 1 | 1 | 1 | 0 | 1 | 84% | HQ |
| Ward et al., 2007 | 1 | 1 | 1 | 1 | 1 | 1 | 1 | 1 | 1 | 1 | 1 | 1 | 1 | 100% | HQ |
| Wasif et al., 2017 | 1 | 1 | 1 | 1 | 1 | 1 | 1 | 1 | 1 | 1 | 1 | 1 | 1 | 100% | HQ |
| Winterbone et al.,2007 | 0 | 1 | 1 | 0 | 0 | 0 | 1 | 1 | 1 | 1 | 1 | 0 | 1 | 61% | MQ |
| HQ: high quality; MQ: medium quality. | | | | | |  |  |  |  |  |  |  |  |  |  |

| **Section and Topic** | **Item #** | **Checklist item** | **Location where item is reported** |
| --- | --- | --- | --- |
| **TITLE** | | |  |
| Title | 1 | Identify the report as a systematic review. | Page 1 |
| **ABSTRACT** | | |  |
| Abstract | 2 | See the PRISMA 2020 for Abstracts checklist. | Page 2 |
| **INTRODUCTION** | | |  |
| Rationale | 3 | Describe the rationale for the review in the context of existing knowledge. | Page 3-4 |
| Objectives | 4 | Provide an explicit statement of the objective(s) or question(s) the review addresses. | Page 3-4 |
| **METHODS** | | |  |
| Eligibility criteria | 5 | Specify the inclusion and exclusion criteria for the review and how studies were grouped for the syntheses. | Page 4 |
| Information sources | 6 | Specify all databases, registers, websites, organisations, reference lists and other sources searched or consulted to identify studies. Specify the date when each source was last searched or consulted. | Page 5 |
| Search strategy | 7 | Present the full search strategies for all databases, registers and websites, including any filters and limits used. | Page 4 |
| Selection process | 8 | Specify the methods used to decide whether a study met the inclusion criteria of the review, including how many reviewers screened each record and each report retrieved, whether they worked independently, and if applicable, details of automation tools used in the process. | Page 5-6 |
| Data collection process | 9 | Specify the methods used to collect data from reports, including how many reviewers collected data from each report, whether they worked independently, any processes for obtaining or confirming data from study investigators, and if applicable, details of automation tools used in the process. | Page 6-7 |
| Data items | 10a | List and define all outcomes for which data were sought. Specify whether all results that were compatible with each outcome domain in each study were sought (e.g. for all measures, time points, analyses), and if not, the methods used to decide which results to collect. | Page 6-7 |
|  | 10b | List and define all other variables for which data were sought (e.g. participant and intervention characteristics, funding sources). Describe any assumptions made about any missing or unclear information. | Page 7 |
| Study risk of bias assessment | 11 | Specify the methods used to assess risk of bias in the included studies, including details of the tool(s) used, how many reviewers assessed each study and whether they worked independently, and if applicable, details of automation tools used in the process. | Page 8 |
| Effect measures | 12 | Specify for each outcome the effect measure(s) (e.g. risk ratio, mean difference) used in the synthesis or presentation of results. | Page 8 |
| Synthesis methods | 13a | Describe the processes used to decide which studies were eligible for each synthesis (e.g. tabulating the study intervention characteristics and comparing against the planned groups for each synthesis (item #5)). | Page 7 |
|  | 13b | Describe any methods required to prepare the data for presentation or synthesis, such as handling of missing summary statistics, or data conversions. | Page 7 |
|  | 13c | Describe any methods used to tabulate or visually display results of individual studies and syntheses. | Page 6-7 |
|  | 13d | Describe any methods used to synthesize results and provide a rationale for the choice(s). If meta-analysis was performed, describe the model(s), method(s) to identify the presence and extent of statistical heterogeneity, and software package(s) used. | Page 7 |
|  | 13e | Describe any methods used to explore possible causes of heterogeneity among study results (e.g. subgroup analysis, meta-regression). | Page 8 |
|  | 13f | Describe any sensitivity analyses conducted to assess robustness of the synthesized results. | Page 8 |
| Reporting bias assessment | 14 | Describe any methods used to assess risk of bias due to missing results in a synthesis (arising from reporting biases). | Page 8 |
| Certainty assessment | 15 | Describe any methods used to assess certainty (or confidence) in the body of evidence for an outcome. | Page 8 |
| **RESULTS** | | |  |
| Study selection | 16a | Describe the results of the search and selection process, from the number of records identified in the search to the number of studies included in the review, ideally using a flow diagram. | Page 9 |
|  | 16b | Cite studies that might appear to meet the inclusion criteria, but which were excluded, and explain why they were excluded. | Page 9 |
| Study characteristics | 17 | Cite each included study and present its characteristics. | Page 9-10 |
| Risk of bias in studies | 18 | Present assessments of risk of bias for each included study. | Page 9-10 |
| Results of individual studies | 19 | For all outcomes, present, for each study: (a) summary statistics for each group (where appropriate) and (b) an effect estimate and its precision (e.g. confidence/credible interval), ideally using structured tables or plots. | Page 10 |
| Results of syntheses | 20a | For each synthesis, briefly summarise the characteristics and risk of bias among contributing studies. | Page 10 |
|  | 20b | Present results of all statistical syntheses conducted. If meta-analysis was done, present for each the summary estimate and its precision (e.g. confidence/credible interval) and measures of statistical heterogeneity. If comparing groups, describe the direction of the effect. | Page 10 |
|  | 20c | Present results of all investigations of possible causes of heterogeneity among study results. | Page 10-11 |
|  | 20d | Present results of all sensitivity analyses conducted to assess the robustness of the synthesized results. | Page 11 |
| Reporting biases | 21 | Present assessments of risk of bias due to missing results (arising from reporting biases) for each synthesis assessed. | Page 11 |
| Certainty of evidence | 22 | Present assessments of certainty (or confidence) in the body of evidence for each outcome assessed. | Figure 2 Figure 3  Figure 4 |
| **DISCUSSION** | | |  |
| Discussion | 23a | Provide a general interpretation of the results in the context of other evidence. | Page 11-12 |
|  | 23b | Discuss any limitations of the evidence included in the review. | Page 13 |
|  | 23c | Discuss any limitations of the review processes used. | Page 13 |
|  | 23d | Discuss implications of the results for practice, policy, and future research. | Page 13 |
| **OTHER INFORMATION** | | |  |
| Registration and protocol | 24a | Provide registration information for the review, including register name and registration number, or state that the review was not registered. | Page 5 |
|  | 24b | Indicate where the review protocol can be accessed, or state that a protocol was not prepared. | Page 5 |
|  | 24c | Describe and explain any amendments to information provided at registration or in the protocol. | N/A |
| Support | 25 | Describe sources of financial or non-financial support for the review, and the role of the funders or sponsors in the review. | Page 14 |
| Competing interests | 26 | Declare any competing interests of review authors. | Page 14 |
| Availability of data, code and other materials | 27 | Report which of the following are publicly available and where they can be found: template data collection forms; data extracted from included studies; data used for all analyses; analytic code; any other materials used in the review. | Page 14 |

*From:*  Page MJ, McKenzie JE, Bossuyt PM, Boutron I, Hoffmann TC, Mulrow CD, et al. The PRISMA 2020 statement: an updated guideline for reporting systematic reviews. BMJ 2021;372:n71. doi: 10.1136/bmj.n71. This work is licensed under CC BY 4.0. To view a copy of this license, visit <https://creativecommons.org/licenses/by/4.0/>
